# Equal access to healthcare in national legislations: how do Croatia, Germany, Poland, and Slovenia counteract discrimination in healthcare?

**DOI:** 10.1186/s12913-021-07453-6

**Published:** 2022-01-24

**Authors:** Katarzyna Bielińska, Anna Chowaniec, Robert Doričić, Marianne Nowak, Marcin Orzechowski, Mojca Ramšak, Paweł Łuków, Amir Muzur, Zvonka Zupanič-Slavec, Florian Steger

**Affiliations:** 1 Center for Bioethics and Biolaw, Faculty of Philosophy, University of Warsaw, Warsaw, Poland; 2 Department of Social Sciences and Medical Humanities, Faculty of Medicine, University of Rijeka, Rijeka, Croatia; 3 Institute of the History, Philosophy and Ethics of Medicine, Ulm University, Ulm, Germany; 4 Institute of History of Medicine, Faculty of Medicine, University of Ljubljana, Ljubljana, Slovenia; 5 Department of Public Health, Faculty of Health Studies, University of Rijeka, Rijeka, Croatia

**Keywords:** Healthcare access, Discrimination, Ethics, National legislation, Croatia, Germany, Poland, Slovenia

## Abstract

**Background:**

The aim of the study was a comparative analysis of legislative measures against discrimination in healthcare on the grounds of a) race and ethnicity, b) religion and belief, and c) gender identity and sexual orientation in Croatia, Germany, Poland and Slovenia.

**Methods:**

We conducted a search for documents in national legal databases and reviewed legal commentaries, scientific literature and official reports of equality bodies. We integrated a comparative method with text analysis and the critical interpretive approach. The documents were examined in their original languages: Croatian, German, Polish, and Slovenian.

**Results:**

All examined states prohibit discrimination and guarantee the right to healthcare on the constitutional level. However, there are significant differences among them on the statutory level, regarding both anti-discriminatory legal measures and other legislation affecting access to healthcare for groups of diverse race or ethnicity, religion or belief, sexual orientation or gender identity. Croatia and Slovenia show the most comprehensive legislation concerning non-discrimination in healthcare in comparison to Germany and even more Poland. Except for Slovenia, explicit provisions protecting equal access for members of the abovementioned groups are insufficiently represented in healthcare legislation.

**Conclusions:**

The study identified legislative barriers to access to healthcare for persons of diverse race or ethnicity, religion or belief, sexual orientation or gender identity in Croatia, Germany, Poland and Slovenia. The discrepancies in the level of implementation of anti-discriminatory measures among these states show that there is a need for comprehensive EU-wide regulations, which would implement the principle of equal treatment in the specific context of healthcare. General anti-discrimination regulations should be strengthened by inclusion of anti-discrimination provisions directly into national legislation relating specifically to the area of healthcare.

## Background

The right to healthcare is tightly connected to the right to health as provided by the Article 12 of the International Covenant on Economic, Social and Cultural Rights of 1966: “the right of everyone to the enjoyment of the highest attainable standard of physical and mental health”. [1] The issue of access to healthcare as a social good sparked the interests of philosophers of social justice in the 1970s focusing on equity in healthcare understood as access to physicians, geographical proximity, and economic status. However, these discussions did not tackle the issue of diversity. Norman Daniels observed that healthcare is a special social good, as health is a condition of equal opportunities. [2] In consequence, he considered healthcare systems as having the moral function “to help guarantee fair equality of opportunity.” [2] (p. 41) This ethical requirement is both descriptive and normative [2] (p. 57).

Later, social philosophers and ethicists shifted from healthcare as the central issue to a broader concept of equity in health, which goes beyond the mere distribution of healthcare. For example, Amartya Sen observed that health equity, is “a multidimensional concept” and ought to be seen as an aspect of the general area of social justice. [3] (p. 26, 31) However, he also said that “nondiscrimination in the delivery of health care” is still of vital importance. [3] (p. 31) Such multidimensional approach was adopted in 2000 by the United Nations Committee on Economic, Social and Cultural Rights, which stated that the right to health is understood “as an inclusive right extending not only to timely and appropriate health care but also to the underlying determinants of health.” [4] The present policy of the European Union (EU) pays special attention to the comprehensive concept of social gradient in health, which means that “people with lower education, a lower occupational class or lower income tend to die at a younger age and to have a higher prevalence of most types of health problems.” [5]

Drawing on Martha Nussbaum’s and Sen’s ideas, J.P. Ruger offers “a capability perspective” on equal access to healthcare, which is not limited to distribution of healthcare, but includes concerns about “healthcare quality, health agency, and health norms.” [6] (p. 92) Ruger claims that when thinking about equal access to healthcare, we should go beyond the horizon of legal norms and focus on public moral norms. [7] However, she acknowledges the primary role of the state in providing “equitable and affordable health care,” [7] (p. 335) and notes the important role of legal measures in this regard, especially in the form of “judicialization of health”, as observed in Latin American states and South Africa. [7] (p. 235). Judicialization of health includes securing access to healthcare goods and services through litigation, which relies on the right to health. In spite of its non-systemic character and even ambiguity, this kind of action can “correct discrimination and unequal treatment.” [7] (p. 235).

Following this approach, we will address the topic of equal access to healthcare in a multi-dimensional way. We will focus on legal measures aimed at combating discrimination in this area based on three dimensions of diversity: a) race and ethnicity, b) religion and belief, c) gender identity and sexual orientation. These dimensions were selected due to their relevance for healthcare. [8]

Equity in access to healthcare and prevention of discrimination in this area in the EU is fragmented because provision and organisation of healthcare remains predominantly in the hands of the Members States. [8] A proposal concerning the implementation of “the principle of equal treatment between persons irrespective of religion or belief, disability, age or sexual orientation” on the EU-level was put forward in 2008, but it has not been adopted yet. [9] It also remains unclear how fragmented the legal situation is, since comparative analyses of the EU member states in this respect are scarce. To begin filling this gap and stimulate further research, we compared the regulatory measures of Croatia, Germany, Poland and Slovenia concerning prevention of discrimination on the basis of the abovementioned grounds. Thus, the main aim of our research was to reconstruct the regulatory landscape of each of the examined states, to reveal potential systemic barriers.

Thus the key research questions we asked were: 1) How have Croatia, Germany, Poland and Slovenia implemented specific European Institutions’ regulations with regard to access to healthcare and anti-discrimination in healthcare into their national legal frameworks? 2) How and to what extent do the national legislations of these four states address access to healthcare and anti-discrimination in healthcare, independently from the EU norms and guidelines? 3) What are the differences among the four states under investigation with respect to access to healthcare and anti-discrimination in healthcare?

These four Member States have been chosen for several reasons. Firstly, they differ with regard to their EU membership duration, with Germany being one of the founding states, Poland and Slovenia joining the EU in 2004, and Croatia in 2013. Secondly, they differ in terms of economic development. Thirdly, their population structures vary in terms of national, religious, and ethnic minorities. Fourthly, and of particular importance from the point of view of this study, the healthcare systems in the four states differ to a large extent. The German healthcare system is based on the long tradition of the Bismarck model executed in the federal state of united lands; Croatia and Slovenia underwent reforms after they became independent from former Yugoslavia, and Poland experienced reforms after the collapse of real socialism. These are single (Croatia, Poland, and Slovenia) or multi-payer (Germany) systems based on obligatory health insurance. [10–13] In Germany, co-payments are relatively limited. [13] In Croatia and Slovenia they are relatively high and can be avoided by purchasing of complementary voluntary health insurance (VHI). [10, 12, 14] In Poland, medicines and other medical goods are co-paid [11] with VHI making healthcare services available outside the public system on a market basis but not for market prices. [14] In Croatia and Poland, the public and private healthcare sectors coexist, with medical workers working for both sectors. [10, 11]

## Methods

We integrated the comparative method with text analysis and critical interpretive approach. The conceptual reference point is the concept of “government-guaranteed equal access to healthcare for all.” [6] (p. 80) “Equal access” means here equal access to adequate goods and services for all persons with diverse healthcare needs who reside on the territory of the examined state, regardless of their formal status [15].

The analysis was designed as documentary research based on national legal documents from all four countries. The documents were identified in the national legislative collections: “Zakon.hr” for Croatia, “Gesetze im Internet” for Germany, “Legalis” for Poland, and “PISRS” for Slovenia. A core set of keywords was created: health, healthcare, discrimination, religion, belief, sexual orientation, ethnicity, gender, sex, minorities. The list was translated and adapted to the four languages (Croatian, German, Polish, and Slovenian) with respect to their linguistic specificities, and supplemented and modified as needed. The search was performed either with single keywords from the list or with their combinations. The search was accompanied by examination of legal commentaries, scientific literature and official reports of appropriate bodies and institutions concerning equality in healthcare, to supplement and verify the results and to provide a comprehensive understanding of and context for the issue under consideration. The documents have been analysed in their original languages: Croatian, German, Slovenian, and Polish.

## Results

The right to healthcare, as a positive entitlement, is constitutionally guaranteed in all examined states [16–22] and finds its expression in legislations on healthcare systems in all examined states, based on insurance and providing specific measures for certain vulnerable groups, such as pregnant persons, persons with disabilities or minors. [23–27] However, those provisions are not derived from contemporary non-discrimination concepts but are rather related to the idea of protection of the weaker.

Our results show that the issue of equity in access to healthcare in the four countries can be analysed from two conceptually different perspectives: the perspective of the principle of equal treatment and prohibition of discrimination, that is, the perspective of human freedoms, or negative rights, and the perspective of the right to healthcare as a social entitlement, or positive right. While the former is regulated by anti-discrimination laws, the latter finds its expression in legislation on healthcare.

These aspects can intersect, which could potentially result in creating conditions of healthy functioning for all individuals, irrespective of their characteristics. With regard to the quality of healthcare, individual’s agency, and societal norms, this intersection could ensure health capabilities, as understood by Ruger [6, 7].

Implementation of anti-discriminatory regulations in healthcare is required only by the Council Directive 2000/43/EC of 29 June 2000 implementing the principle of equal treatment between persons irrespective of racial or ethnic origin. [28] Therefore, all examined states have relevant provisions in their anti-discriminatory regulations. Member states are obligated to implement the EU Directives in their minimum, but they can also decide if they will guarantee more comprehensive protections or not, and what specific part of their legislations will implement them. This, in turn, can translate into an impact on the legislation on a particular sector of social life. We assume that protections against discrimination in healthcare will tend to be strongest and potentially most effective when the provisions protecting equal treatment of diverse groups are included directly into the regulations on healthcare rather than merely in general anti-discrimination laws. We analysed our results from those two perspectives. Additionally, having observed the lack of provisions concerning cultural competency on the part of healthcare workers, we found out that regulations addressing religion and independent from the EU norms and guidelines are of special importance. We have also identified various legal barriers in access to healthcare as such, to specific services, or for certain groups such as migrants.

### Implementation of the European anti-discrimination norms and guidelines

All constitutions of the examined states guarantee equal treatment and prohibit discrimination. However, their provisions vary with regard to the range of potential grounds of discrimination explicitly recognized. The Croatian and Slovenian basic laws provide long lists of exemplary grounds, on which discrimination is prohibited: “race, colour, gender, language, religion, political or other opinion, national or social origin, property, birth, education, social status or other status” (CoC, Art. 14) and “national origin, race, sex, language, religion, political, or other conviction, material standing, birth, education, social status, disability, or any other personal circumstance” (CoS, Art. 14). The Article 3 p. 3 of the Germany’s Basic Law prohibits discrimination on the basis of “sex, parentage, race, language, homeland and origin, faith or religious or political opinions and disability.” In Poland, “No one shall be discriminated against in political, social or economic life for any reason whatsoever” (CoP, Art. 32 p. 2). The German and Polish basic laws contain separate provisions on the equality of men and women (BLFRG, Art. 2.2, CoP Art. 33). Constitutional documents of the examined states guarantee freedom of religion. Furthermore, they specifically address equal treatment for recognized ethnic and national minorities.

Such constitutional guarantees are followed by anti-discriminatory legislations which implement the EU equal treatment directives. Regarding race and ethnicity, religion and belief, and sexual orientation and gender identity, the comparative analysis reveals deep discrepancies. Anti-discrimination legislations are not applicable by default to all areas of social and public life. These were initially introduced in the area of labour law and have been later extended to other areas. On the European level, the area of healthcare is still only partly addressed. Thus, for the purpose of our study, it was crucial to identify the potential grounds of discrimination recognized by anti-discrimination legislation, in relation to healthcare.

Chronologically first was the General Act on Equal Treatment in Germany (2006). It prohibits discrimination on “the grounds of race or ethnic origin, gender, religion or belief, disability, age or sexual orientation” (Sec. 1) and explicitly addresses the area of healthcare (Sec. 2, p. 5). [29] In so doing, the German Act on Equal Treatment goes beyond the minimum required by the EU. This enumerated list of grounds of discrimination does not include gender identity. However, in Germany, the concept of gender is understood broadly and includes gender identity. Therefore, although this legal act does not cover all examined grounds literally, it does so in practice.

In Poland the so-called Equality Act (2010) lists “sex, race, ethnic origin, nationality, religion, denomination, beliefs, disability, age or sexual orientation” (Art. 1). [30] However, Article 7 specifies grounds of protection in the area of healthcare as “race, ethnic origin or nationality”, thereby adding nationality to the required minimum. Therefore, in case of discrimination in healthcare on the basis of religion or belief or sexual orientation or gender identity the Equality Act cannot be cited as the legal basis of complaint. In such cases one can only seek appropriate legal remedy for infringement of their personal rights.

The Croatian Anti-Discrimination Act (2008, amended 2012) provides an enumerated list of protected grounds: “race or ethnic affiliation or colour, gender, language, religion, political or other belief, national or social origin, property, trade union membership, education, social status, marital or family status, age, health condition, disability, genetic heritage, gender identity, expression or sexual orientation” (Art. 1) and addresses the health insurance and healthcare (Art. 8 p. 3 and 4). [31] Thus, complaints regarding discrimination in healthcare can be made according to dedicated procedures.

The Slovenian Protection Against Discrimination Act (2016, amended 2018) provides an exemplary list of grounds: “gender, nationality, racial or ethnic origin, language, religion or belief, disability, age, sexual orientation, gender identity or gender expression, social status, property status, education, or any other personal circumstance” (Art. 1). [32] This act also addresses healthcare explicitly (Art. 2 p. 1). Since this catalogue is accompanied by the phrase “or any other personal circumstance”, which is open to interpretation, the act can cover much broader scope of discrimination cases than the Croatian law.

Thus, protections against discrimination in healthcare are most comprehensive in Slovenia and Croatia. The German General Act on Equal Treatment does not explicitly recognize gender identity but it does apply to it. The so-called Equality Act in Poland lags behind all of them, by leaving out discrimination in healthcare on the basis of religion and belief, or sexual orientation and gender identity.

The EU does not require member states to legally recognize non-heteronormative partnerships. Accordingly, the situation of non-heteronormative couples with regard to access to healthcare differs among the examined countries. While legislations on same-sex partnership in Croatia and Slovenia, and marriage equality in Germany guarantee access to healthcare for non-heteronormative partners on the basis of a partner’s health insurance, in Poland, whose law does not recognize non-heteronormative couples, there is no such explicit entitlement. [33, 34]

### National legislation on healthcare

Legislations on healthcare can protect patients’ rights, provide institutional solutions or establish legal apparatus. However, with the exception of Slovenia, the states under consideration are reluctant to incorporate into their healthcare legislations explicit anti-discrimination regulations regarding the grounds under investigation.

#### Patients’ rights acts

Patients’ rights acts have been adopted by all examined states. They cannot be easily classified as protecting individual rights and freedoms or guaranteeing social rights, as they combine both aspects. They can play the role of a legal instrument, which transposes protection against discrimination into the sphere of healthcare. These acts establish patients’ rights offices (such as ombudsperson office), in order to facilitate claims in case of discrimination in healthcare.

Legislations on patients’ rights protect freedoms of patients, such as the right to privacy, confidentiality, intimacy, consent and information, and address the issue of equality in healthcare to various extent. The Act Improving the Rights of Patients in Germany (2013) does not refer directly to non-discrimination and equal access. [35] In Croatia the Act on the Protection of Patients’ Rights (2004, with amendments) [36], in Poland the Act on Patient’s Rights and Patient’s Rights Ombudsman (2008, with amendments) [37] and in Slovenia Patients’ Rights Act (2008, amended 2017) [38], guarantee the right to proper and equally accessible medical treatment. Only the Slovenian act contains a provision prohibiting discrimination on exemplary grounds: “sex, nationality, racial or ethnic origin, religion or belief, disability, sexual orientation or any other personal circumstance” (Art. 7). However, Croatian and Polish acts address specifically patients’ religious freedoms. In Croatia, Poland and Slovenia patients whose rights have been violated can complain to ombudspersons or to patients’ rights offices, such as the Regional Commissions for Patient’s Rights Protection and Ministerial Commission for Patient’s Rights Protection in Croatia, Patient’s Rights Ombudsman in Poland, and Patients’ Rights Advocates in Slovenia, or to general ombudspersons.

Acts on patients’ rights often refer to the ethical principles, which are prescribed by the respective professional legal acts and codes of ethics. Such documents prohibit discrimination and require medical staff members to provide care regardless of personal circumstances. [39–41] However, ethics codes do not have the same status as legislative acts and therefore cannot be the basis for legal action. They can, however, play a supplementary role. Discrimination claims can be filed to professional responsibility bodies, if not guaranteed by other legislative acts.

#### Legislation addressing access to healthcare in specific areas

Access to healthcare for persons of diverse race or ethnicity, religion or belief, or sexual orientation or gender identity is also affected by legislation that addresses provision of specific healthcare goods and services with the notable example of sexual and reproductive health goods and services. For example, in all four countries under investigation, non-heteronormative persons are denied access to medically assisted procreation, which is available only to persons in heteronormative relationships [27, 33, 34, 42–44]. States also vary with regard to the accessibility of gender confirmation services in relation to both payment and administrative barriers.

Specific provisions of other legislative acts also affect access to healthcare for various groups. Regulations on the relations between the state and religious communities (such as concordats), specifically address access to pastoral/spiritual care and freedom of religious practices in medical facilities. Legislations on national and ethnic minorities aim to meet their special needs, including in the area of healthcare [45, 46]. Those legislations are followed by further regulations or programs, e.g. National Roma Inclusion Strategy from 2013 to 2020 in Croatia, the Multiannual Programme for the Integration of the Roma Community for 2014–2020 in Poland, or National Program of Measures of the Government of the Republic of Slovenia for Roma for the Period 2017–2021 [47–49]. Such regulations are of particular nature. They concern members of specific, historically present communities, and so they can produce inequalities between “old” and “new” (i.e. migrant) minorities.

#### Access to healthcare for migrants and persons without entitlements

Access to healthcare for EU-citizens in each of the examined states is guaranteed on the basis of social security coordination and the legislation implementing the Directive 2011/24/EU. [50] Access to healthcare for non-EU citizens is regulated by respective legislation on foreigners. In principle, in all examined states, persons who are legally employed or have refugee status or international protection, are provided with access to healthcare according to the same rules as citizens.

For asylum-seekers, access to healthcare is regulated separately. Croatia, Germany and Slovenia have lists of services guaranteed to asylum-seekers. However, they include only a small portion of  the services guaranteed to people with health insurance. In Croatia asylum-seekers have guaranteed access to emergency care, treatment of urgent cases, and serious mental disorders; additional care is provided to persons who have experienced severe forms of violence. [51] In Germany, asylum-seekers are entitled to treatment in case of acute illness or pain, vaccination, medically indicated preventive check-up, obstetrics, child-delivery and post-partum care, and urgent dental services in individual cases. Those who remain in the country longer than 18 months during their application process, are provided with the same scope of services as insured citizens [52]. In Slovenia, the free care package for asylum-seekers includes emergency medical and dental care, sexual and reproductive health services together with contraception and abortion; minors enrolled in the school system and students under 26 are granted the same entitlements as citizens. Additional care is provided to vulnerable persons [53].  In Poland, the package of medical services provided to the applicants for the refugee status or protection is formally the same as for persons holding public health insurance, with the exception of spa treatment and spa rehabilitation. Given this, in practice, the implementation of this formal entitlement could be limited by the fact that healthcare for asylum seekers is provided on the basis of agreements between the Head of the Office for Foreigners and healthcare providers rather than within the general healthcare system. [54]

Undocumented migrants in Croatia, Poland and Slovenia are seen by the systems as persons without entitlements to public healthcare system. Therefore, they can obtain the minimum of services that is provided to everyone without payment regardless of their legal status, insurance or other formal claims. This minimum can be different in different countries. In Croatia there are no unpaid services for undocumented migrants. In Poland they have free access to some services, including emergency care in outpatient settings, treatment of infectious diseases, or HIV testing and counselling. [55–57] In both countries they can purchase healthcare services for market prices, if they can afford them. In Slovenia undocumented migrants have access to primary healthcare in pro bono clinics. In Germany the situation of undocumented migrants differs from other uninsured persons. Formally, undocumented migrants are entitled to the same healthcare package as asylum-seekers. In practice, however, seeking this help is burdened with a substantial risk of deportation, because healthcare facilities belong to the entities which are required to notify the police if they have information about undocumented migrants.

#### Healthcare legislation – in general

Diversity as an integral element of the healthcare system has been explicitly identified only in Slovenia. Such integration is a condition of access to healthcare as understood by the capability theory. In this way it is responsive to the diverse needs of patients, and such responsiveness is often conceptualized as cultural competency. However, with the exception of some elements addressing religious freedoms or minority rights of historically rooted communities, we have not identified in the analysed documents provisions which would specifically address cultural competences.

## Discussion

According to J.P. Ruger, the goal of the comprehensive approach to the issue of access to healthcare is the full actualization of individual health potential. Following this interpretation, adequate attention must be paid to culturally sensitive healthcare. [7] This idea originally referred to race and ethnicity. [58] Later, it has been broadened to encompass religion, sexual orientation and gender identity. [59] Cultural sensitivity is linked with protection against discrimination and guarantees of equal access for groups under discussion. [60]

### Race and ethnicity

It has been observed that “the systematic neglect of culture in health and health care is the single biggest barrier to the advancement of the highest standard of health worldwide.” [61] (p. 1610). In Europe, in the context of the recent transformation of diversity, also called super-diversity, new challenges have emerged for healthcare. [62] This concept, proposed by Vertovec, addresses the new socio-cultural landscape with new patterns of migration and “new hierarchical social positions, statuses or stratifications.” [63] (p. 121) It has been adopted in health studies, along with the observation of the need for redefinition of the concept of cultural competences in healthcare and a shift from cultural characteristics to relations of power [64, 65].

Although all examined states have implemented the prohibition of discrimination on the grounds of race and ethnicity in their anti-discrimination legislations, it is just a condition for creating culturally competent healthcare concerning these categories.

Compared to other countries discussed here, Germany has the most advanced form of culturally sensitive healthcare [66]. However, also in this country members of diverse groups, such as migrants and asylum-seekers encounter obstacles in their access to healthcare [67]. Those barriers are not addressed sufficiently by the existing legislation.

Legal limitations of access to healthcare can provoke a conflict between medical professional ethics and legal regulations [68]. Undocumented migrants are in a particularly difficult position. The comparison of the examined states has shown that there is no uniform pattern of healthcare services for undocumented migrants, which corresponds with other studies [69]. Even when they are granted access to healthcare, migrants and ethnic minorities may encounter other barriers, such as language and culture [70]. Special needs of ethnic minorities, e.g. Roma, are addressed by several projects in the scope of governmental and European programmes [71]. However, except for the Slovenian Patients’ Rights Act and Health Care and Health Insurance Act, their rights are not specifically addressed by the legislation on healthcare, although all states under examination have implemented the provisions about protection against discrimination on the grounds of race or ethnic origin into their general anti-discrimination laws.

### Religion and belief

By contrast, with regard to equal access to healthcare regardless of religion and belief, our results confirm that an important role in this field is played both by regulations on the relations between the state and religious communities and by guarantees in legislation addressing healthcare. This creates a complex picture. The right to pastoral/spiritual care is guaranteed in various ways, with some religious communities being privileged over others. [72] However, in the examined states, rights of members of diverse religious communities are guaranteed to a relatively large extent. Introduction of specific provisions into the legal acts that address healthcare, directly identifies the facilities that are obligated to follow certain rules. [73]

### Sexual orientation and gender identity

Non-heteronormative persons encounter numerous barriers in their contacts with healthcare and appropriate protections in removing these barriers play an important role. [74] The described level of protection could be significantly undermined by political and ideological trends, such as with the infamous example of “LGBT-free zones” in Poland. [75] Meeting specific healthcare needs of non-heteronormative persons is interconnected with general access to sexual and reproductive health goods and services. The latter is subject to ideological motivations and reproductive governance which could lead to violations of sexual and reproductive rights, like in Poland and Croatia. [76, 77]

The lack of sufficient protection of non-heteronormative persons in healthcare leads to a situation, in which they must claim their rights on the basis of other legal guarantees, such as on the right to privacy, confidentiality, intimacy, consent, or information, as in Poland. [78] Access to certain goods and services could be limited for transgender persons due to gendering in healthcare, which finds expression in legal formulations, e.g. providing gynaecological or obstetric care for women. [79] Therefore it is important to address the issue explicitly by law, as in Croatia or Slovenia.

A clear example of the legal discrimination on the basis of sexual orientation and gender identity, which could be derived from the aforementioned national legislation, is the denial of access to medically assisted procreation to non-heteronormative persons. Such an approach has been identified as discriminatory both by the American Medical Association, U.S. case law, and literature. [80, 81] Gender confirmation treatment is an issue which raises various concerns regarding equal access to healthcare for non-heteronormative persons. [82]

### Limitations

Limitations of the present study stem from the character of the material under investigation. It includes not only national regulations, implementing the European Union general norms and guidelines, but also provisions regarding access to healthcare for minority groups which are contained in various national legislations embedded in national contexts. While the former rely on one conceptual apparatus, the latter use their own conceptualisations. This difference is relevant in that it would often be presumptuous to evaluate comparatively developments and implications of those complex normative frameworks.

Since our aim was to provide the evaluation of equality in access to healthcare in the European perspective, with the focus on the perspectives of the states under consideration, we adopted a more comprehensive approach that includes a range of legal acts. This results in a more complex and adequate picture, which, however, makes it more difficult to draw unequivocal conclusions. Thus, there are three more specific limitations of conceptual nature.

The first relates to race and ethnicity. Numerous legislations on national and ethnic minorities’ or foreigners’ rights overlap with, but do not fit neatly into, the concepts appropriated from the EU legal discourse. Also, except for international legislation, the concept of migrant is rather absent from the legal systems of Poland, Croatia and Slovenia. Unlike Germany, those states do not have dedicated legislations on immigration but rely on the concepts of “aliens” or “foreigners”.

The second limitation, one related to religion and belief, lies in the fact that numerous dedicated legislations and provisions guarantee rights and entitlements to specific religious communities, and do not have a general status. It is thus difficult to draw more comprehensive conclusions in relation to this issue.

The third limitation stems from the focus on the concept of gender identity, leaving out sex/gender. This focus is justified by the recognition that legislation relies significantly on the binary biological concept of gender, often providing specific entitlements to “women”, “mothers” or “pregnant women”.

The limitations mentioned above are enhanced by the fact that general legal frameworks of the states under consideration also vary due to historical reasons. The legal acts that fall within the scope of our study sometimes precede national basic laws. Thus, apart from the general picture based on the elements of diverse conceptual schemes, which is offered here, an in-depth investigation of institutional regulations is needed. This is the topic of our further research.

## Conclusions

There are two general conclusions of our analysis:

1. We believe that EU directives are the most efficient way of harmonizing national legislations. However, the only directive, which explicitly addresses healthcare, is Directive 2000/43/EC. A Directive which would address discrimination on other grounds than race and ethnicity in non-occupational areas, including healthcare, has not been adopted yet. Therefore, states are free to decide on their own what other anti-discriminatory laws they have in this area. Also, healthcare as such remains mainly in the hands of the Members States. In effect, it is national legislations that shape the field of equal access to healthcare and protection against discrimination.

Thus, the legislation on the protection against discrimination in healthcare on grounds other than race and ethnicity differs significantly among states. With regard to such categories as religion and belief, sexual orientation, and gender identity, such divergences exist among Croatia, Germany, Poland and Slovenia. The relevant legislations are most comprehensive in Slovenia and Croatia, then in Germany with Poland lagging behind. Because of this, individuals from minority groups may experience barriers in access to healthcare goods and services, despite the principles of equality, which are clearly stated in the treaties and documents of the European Union.

2. Anti-discrimination legislation is, however, just the first step on the way to equal access to healthcare. Health capability oriented and culturally competent healthcare, which would respond adequately to diverse patients’ needs, must be organized according to principles of non-discrimination. Currently, access to healthcare for members of various communities is determined, directly or indirectly, by other regulations, embedded mostly in national contexts, sometimes privileging historically rooted communities over the “new” ones. Therefore, the regulatory landscape is complex, with numerous barriers to be expected to emerge.

We thus suggest that comprehensive anti-discrimination regulations are built into healthcare legislations to help to overcome this situation. Broad anti-discrimination provisions built directly into legislations on healthcare would encourage institutions to introduce systemic measures of protection against discrimination against patients of diverse characteristics.

Therefore, we recommend, first, adoption of a general anti-discrimination Directive on the EU level, that would apply to all states and require them to implement the equal treatment principle in the context of healthcare regardless of, among others, religion or belief, gender identity or sexual orientation. Secondly, we recommend introduction of broad anti-discrimination provisions directly into legislations on healthcare.

## Data Availability

The datasets generated and/or analysed during the current study are available in: “Zakon.hr” for Croatia—https://zakon.hr/ “Gesetze im Internet” for Germany—https://www.gesetze-im-internet.de/ “Legalis” for Poland—https://legalis.pl/ “PISRS” for Slovenia—http://www.pisrs.si/Pis.web/

## Abbreviations

BLFRG: Basic Law for the Federal Republic of Germany
CoC: Constitution of the Republic of Croatia
CoP: Constitution of the Republic of Poland
CoS: Constitution of the Republic of Slovenia
EU: European Union
VHI: Voluntary Health Insurance
